# Induction of ER stress-mediated apoptosis by ceramide via disruption of ER Ca^2+^ homeostasis in human adenoid cystic carcinoma cells

**DOI:** 10.1186/2045-3701-4-71

**Published:** 2014-11-25

**Authors:** Zhe Liu, Yichao Xia, Bo Li, Hui Xu, Chenxing Wang, Ying Liu, Yi Li, Chunjie Li, Ning Gao, Longjiang Li

**Affiliations:** Department of Head and Neck Oncology, West China Hospital of Stomatology, Sichuan University, Chengdu, PR China; State Key Laboratory of Oral Diseases, West China Hospital of Stomatology, Sichuan University, Chengdu, PR China

**Keywords:** Ceramide, ER stress, ER calcium, Apoptosis, Cancer, Unfolded protein response

## Abstract

**Background:**

Ceramides are a class of sphingolipids that form the structural component of the cell membrane and also act as second messengers in cell signaling pathways. Emerging results suggest that ceramide induces growth arrest and apoptosis in various human cancer cells. However, the mechanisms underlying its antitumor activity are yet to be identified. Endoplasmic reticulum stress (ER stress), a cellular adaptive response, is believed to initially compensate for damage but can eventually trigger cell death if the stimulus is severe or prolonged. In this study, we investigated whether ceramide induces cell death in human salivary adenoid cystic carcinoma (ACCs) through activation of the apoptotic ER stress.

**Results:**

RT-PCR, real-time PCR and western blot demonstrated that exogenous ceramide treatment up-regulated GRP78 and p-eIF2α expression and XBP1 splicing. Moreover, the ceramide synthase inhibitor FB1 abolished ceramide-induced ER stress. Up-regulation of the ER stress-associated apoptosis promoting transcription factor CHOP and p-JNK suggested that the antitumor activity of ceramide is owing to activation of apoptotic ER stress. Mechanistically, [Ca^2+^]_ER_ depletion and SERCA inhibition by ceramide treatment suggested that it induces ER stress by disrupting [Ca^2+^]_ER_ homeostasis. The chemical chaperone TUDCA inhibited ceramide-induced ER stress and cell death. In addition, the downstream metabolite of ceramide, S1P, cannot activate ER stress.

**Conclusions:**

These results demonstrated that exogenous ceramide induces cancer cell death through a mechanism involving severe ER stress triggered by the disruption of ER Ca^2+^ homeostasis.

## Introduction

Rapid proliferation of cancerous cells during cancer progression places a high demand on protein synthesis. The endoplasmic reticulum (ER) is a critical organelle in the synthesis, proper folding and assembly of secretory and membrane proteins [1]. It is also the site of lipid synthesis and a major intracellular Ca^2+^ reservoir. Cellular stimuli that perturb ER homeostasis, including hypoxia, failure of protein synthesis, folding, transport or degradation, ER Ca^2+^ depletion and oxidative stress, may lead to ER stress. ER stress triggers the surveillance mechanism known as the unfolded protein response (UPR). The UPR involves the activation of inositol-requiring protein 1α (IRE1α), PKR-like ER kinase (PERK) and activating transcription factor 6 (ATF6). Activation of the UPR minimizes ER stress by improving the protein folding capacity of the ER, halting the rate of secretory protein synthesis and increasing the chaperone capacity in cells.

However, persistent or severe ER stress activates a UPR that results in apoptosis. Activated PERK, IRE1α and ATF6 under chronic ER stress regulate downstream targets, mainly the CCAAT/enhancer-binding protein (C/EBP) homologous protein (CHOP) and JNK, which play important roles in the commitment phase of ER stress-mediated apoptosis. CHOP inhibits expression of the anti-apoptotic protein Bcl-2 and induces the expression of the pro-apoptotic Bcl-2 family member Bim [2–4]. Activation of either IRE1α-TRAF2-ASK1 or CHOP-CAMK II pathways under ER stress induces JNK phosphorylation, which activates ER stress-mediated apoptosis through at least two mechanisms: induction of Fas and induction of Nox2 and subsequent oxidative stress [5–7]. Overwhelming ER stress eventually activates apoptosis through cleavage of caspase-12 in murine cells or caspase-4 in human cells, which subsequently activates executioner caspases such as caspase-3 [8–10].

The interconvertable sphingolipid metabolites, ceramide and sphingosine-1- phosphate (S1P), constitute the sphingolipid rheostat. The dynamic balance of these two constituents has long been proposed to control the fate of the cell; with S1P promoting cell growth and survival, whereas ceramide drives apoptosis, autophagic responses and cell cycle arrest [11, 12]. Ceramide is produced by ceramide synthase through *de novo* biosynthesis in the ER. Recent studies suggested that alteration of ceramide synthase 6 (CerS6) activates the ATF6-CHOP arm of the UPR pathway and induces apoptosis [13, 14]. It was also reported that the combined treatment of sorafenib and vorinostat induces ER stress and apoptosis through elevation of ceramide level and CD95 activation [15]. However, the mechanisms by which exogenous ceramide regulates ER stress and subsequent apoptosis remain unknown.

In this study, we have identified that exogenous ceramide triggers an apoptotic ER stress response by treating salivary adenoid cystic carcinoma cells (ACCs) with cell-permeable short chain C2-ceramide. We defined a novel mechanism that activates ER stress via SERCA inhibition and [Ca^2+^]_ER_ depletion in response to ceramide treatment. Furthermore, we observed that ceramide induces apoptosis via activation of pro-apoptotic factors downstream of ER stress in ACCs.

## Results

### Ceramide induces sustained activation of XBP1 mRNA splicing in ACCs

**Figure 1:**
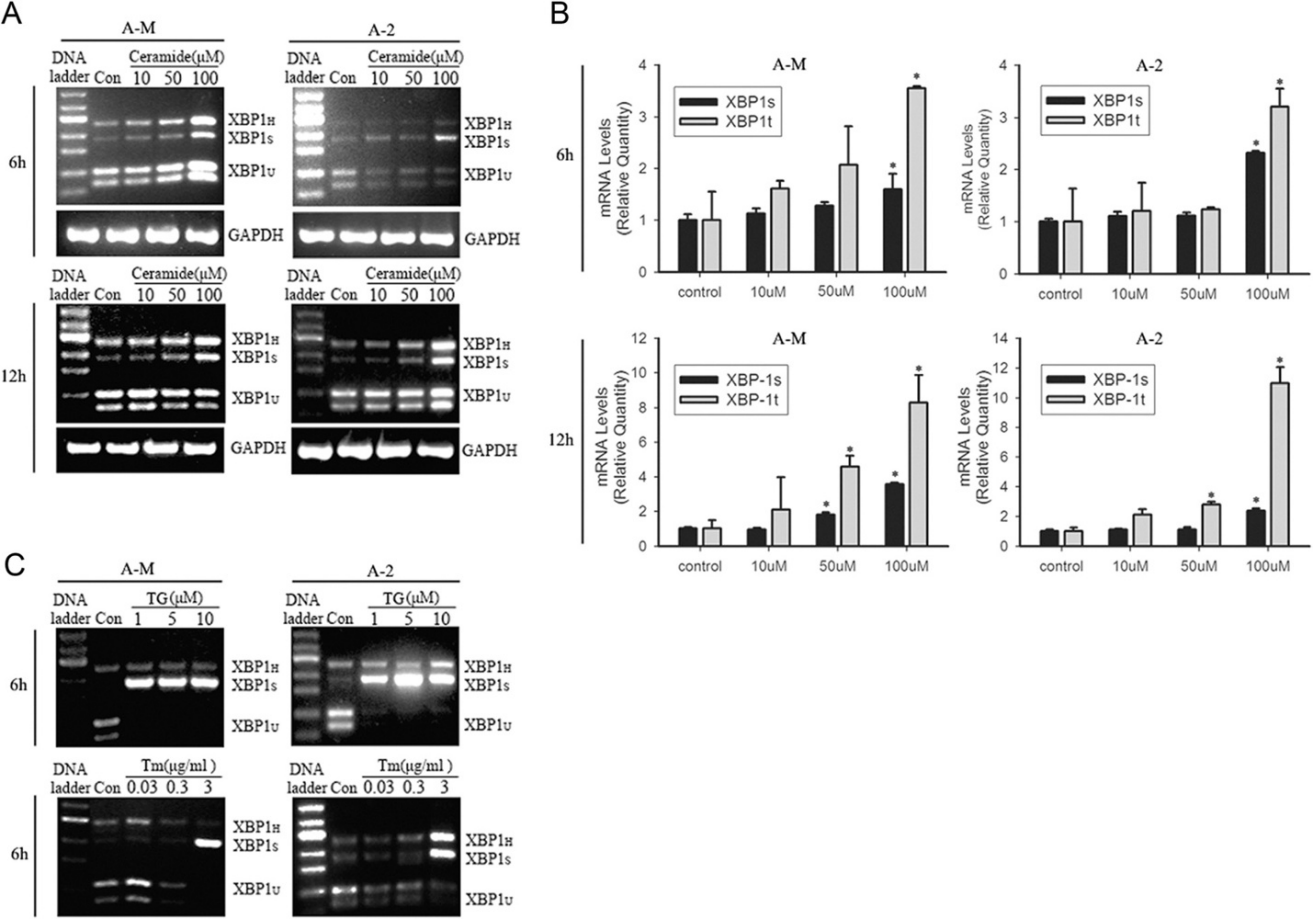
**Ceramide activates XBP1 mRNA splicing in ACCs. (A)** ACC-M or ACC-2 cells were subjected to the indicated concentration (10–100 μM) of C2-ceramide, and the total RNA was isolated 6 and 12 h after ceramide treatment. Reverse transcription to cDNA and RT-PCR was performed to detect spliced (XBP1_S_) and unspliced (XBP1_U_) forms of XBP-1. GAPDH was used as a loading control. RT-PCR products were digested by PstI restriction enzyme in 37°C for 1 h, and then separated on 2% agarose gel and visualized in an UV image system. **(B)** Cells were treated as in A, and the total RNA was subjected to Real-time PCR. Relative mRNA expression levels for XBP1_S_ and XBP1_U_ were calculated by normalizing to the signal for GAPDH mRNA in each sample and comparison with cells cultured in a control medium. The figure presents mean fold over control change in experimental groups ± S.D. *P < 0.05 (one-way ANOVA). **(C)** Cell were treated with 1–10 μM Thapsigargin or 0.03-3 μg/ml Tunicamycin (positive control for XBP1_S_ expression), RT-PCR for XBP1 mRNA splicing detection was performed as in A.

To test the hypothesis that ceramide acts as an ER stress activator in ACCs, exogenous cell permeable short chain C2-ceramide was used to treat ACC-M and ACC-2 cells. Reverse Transcription-PCR (RT-PCR) showed a significant increase in the expression of the spliced isoform of XBP1 (XBP1_S_) after treatment with 100 μM ceramide for 6 h, and prolonged incubation with ceramide for 12 h further increased this effect (Figure 1A). Changes in XBP1 mRNA splicing were detected by RT-PCR amplification, followed by PstI digestion. There is a PstI site in the 26-nucleotide intron of XBP1_U_ but not in XBP1_S_ mRNA. Digestion of the RT-PCR products with a PstI restriction enzyme enables XBP1_S_ (not digested) and XBP1_U_ (digested into two smaller bands) to be distinguished [16]. We also observed a slowly migrating species (XBP1_H_), which represents a hybrid structure of unspliced and spliced single stranded DNAs [17, 18]. Real-time PCR confirmed that ceramide induced XBP1 mRNA splicing in a time- and dose-dependent manner (Figure 1B). Tunicamycin (Tm) and Thapsigargin (TG) are classic inducers of ER stress, and were used as positive controls in our study. Tunicamycin inhibits protein glycosylation in the ER, leading to protein misfolding and subsequent ER stress. Thapsigargin selectively inhibits sarcoplasmic/endoplasmic reticulum Ca^2+^- ATPase (SERCA), resulting in Ca^2+^ depletion from the ER lumen and activation of ER stress. RT-PCR showed that treatment with 1–10 μM TG or 3 μg/ml Tm for 6 h significantly induced XBP1 mRNA splicing (Figure 1C).

### Ceramide activates eIF2α phosphorylation and increases GRP78 expression

**Figure 2:**
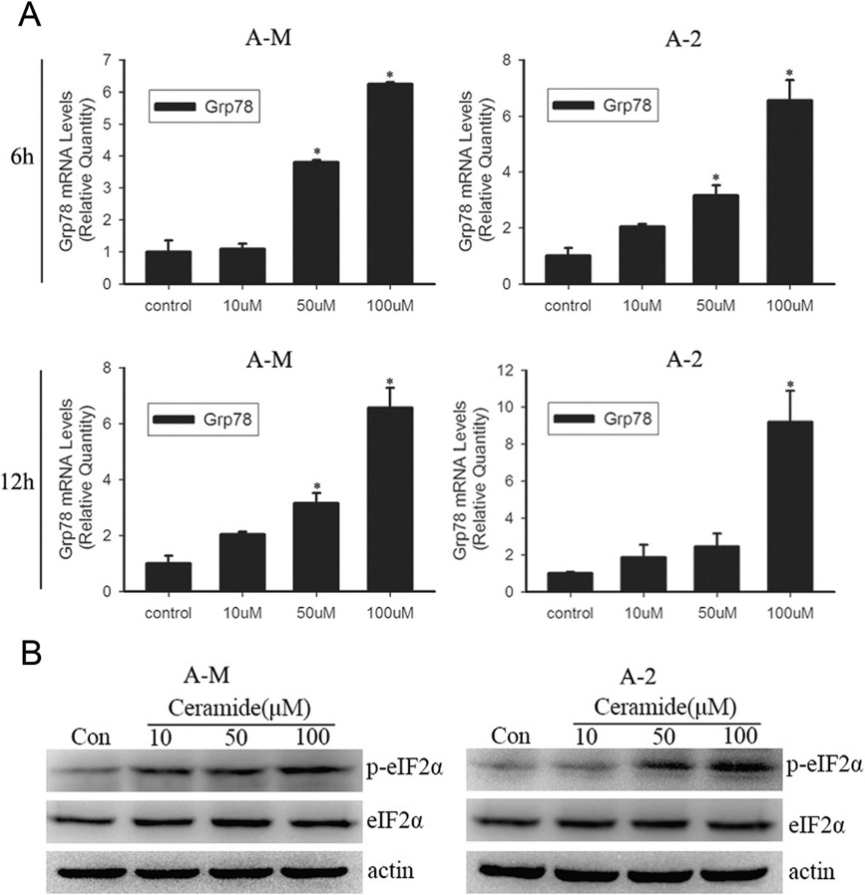
**Ceramide markedly increases Grp78 mRNA expression and eIF2α phosphorylation. (A)** ACC-M and ACC-2 cells were treated with indicated concentration (10–100 μM) of ceramide for 6 and 12 h, total RNA was subjected to Real-time PCR. Relative mRNA expression levels for Grp78 were calculated by normalizing to GAPDH. *P < 0.05 (one-way ANOVA) versus control cells. **(B)** Cells were seeded into 60 mm culture dishes and the next day cells were treated with ceramide. After 3 h incubation, whole cell lysates were collected and subjected to western blot. The level of phosphorylated eIF2α was analyzed with anti-phosphorylated eIF2α antibody. Unphosphorylated eIF2α were measured and actin were used as loading controls. The experiment was repeated several times and the representative result is shown.

ER resident protein chaperons such as GRP78 and GRP94 assist in the proper folding, maturation and stabilization of nascent proteins in ER [19]. Elevated GRP78 expression is an indicator of ER stress. We used Real-time PCR to analyze the change in GRP78 expression after ceramide treatment in ACC-M and ACC-2 cells. Incubation with 100 μM ceramide for 6 h significantly increased GRP78 expression. Similar to the expression pattern of XBP1_S_, the prolonged incubation time of 12 h induced higher levels of GRP78 expression (Figure 2A). Upon ER stress, activated PERK phosphorylates eukaryotic initiation factor 2α (eIF2α), which attenuates the overall mRNA translation rate while inducing the translation of selective mRNAs with inhibitory uORFs in their 5′ UTR [20]. Western blot analysis showed increased eIF2α phosphorylation after treatment with 100 μM ceramide for 3 h (Figure 2B). These results suggest that ceramide activates ER stress in a time- and dose-dependent manner in ACCs.

### Inhibition of ceramide synthase by FB1 impairs ceramide-induced ER stress

**Figure 3:**
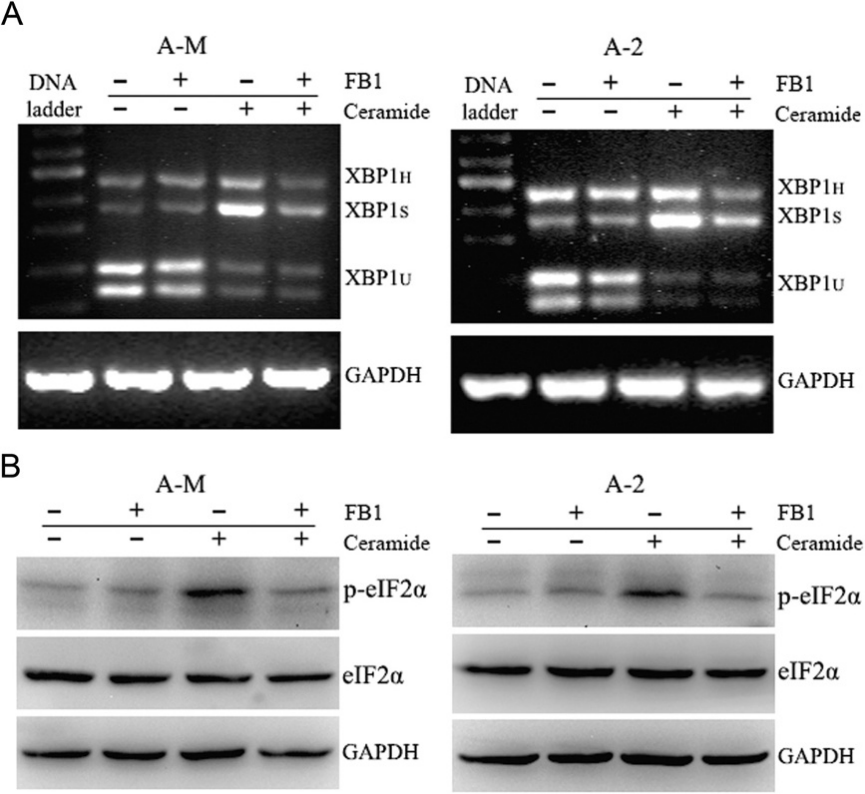
**Fumonisin B1 inhibits ceramide-induced ER stress. (A)** ACC-M and ACC-2 cells were seeded into 60 mm culture dishes and the next day cells were pretreated with 20 μM FB1. After 3 h incubation, 100 μM ceramide was added for further 12 h incubation in full medium. Total RNA was isolated for detection of XBP1 mRNA splicing using RT-PCR. The experiment was repeated three times and the representative result is shown. **(B)** Cells were treated as in A. After 20 μM FB1 treatment for 3 h, 100 μM ceramide was added for further 3 h incubation. Whole cell lysates were collected to analyze phosphorylated eIF2α expression. The experiment was repeated several times and the representative result is shown.

Fumonisin B1 (FB1) is a natural competitive inhibitor of ceramide synthase. Treatment with FB1 inhibits the synthesis of ceramide and its downstream metabolites. We therefore examined the effect of FB1 on ceramide-induced ER stress. RT-PCR showed that 3 h pretreatment with 20 μM FB1 abolished ceramide-induced XBP1 mRNA splicing (Figure 3A). Western blot analysis demonstrated the inhibitory effect of FB1 on ceramide-induced eIF2α phosphorylation (Figure 3B). These results suggest that the ceramide synthase inhibitor FB1 abolishes ceramide-induced ER stress.

### Ceramide induces [Ca^2+^]_ER_ depletion and SERCA inhibition leading to ER stress

**Figure 4:**
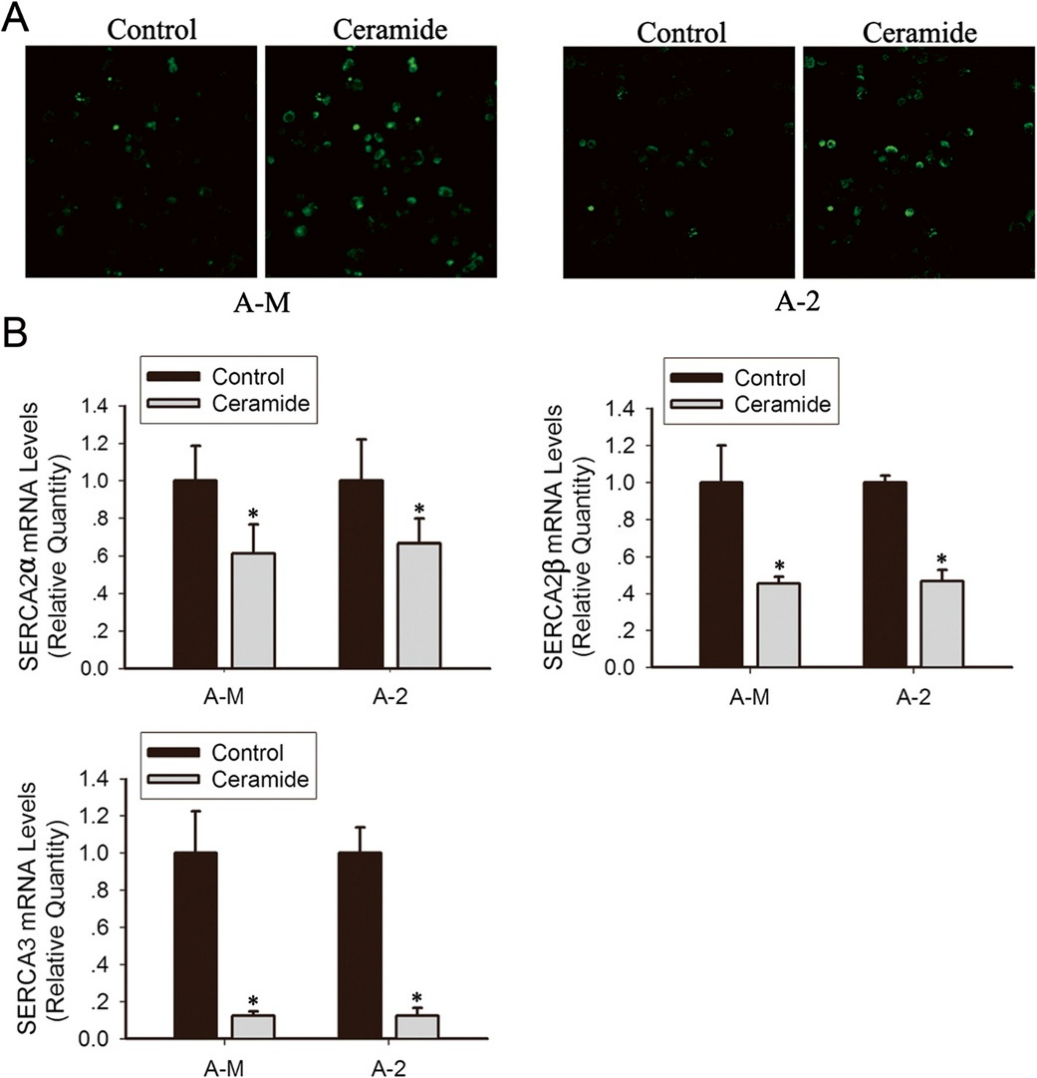
**Ceramide mediates [Ca** ^**2+**^ **]**_**ER**_ **depletion through inhibition of SERCA expression. (A)** ACC-M and ACC-2 cells were loaded with Ca^2+^-sensitive probe Fluo 4-AM. Changes in fluo-4 fluorescence were determined using confocal video microscopy. As shown in the representative images, perfusion with 100 μM ceramide induced increases in [Ca^2+^]_ER_ depletion in ACCs. **(B)** Cells were treated with 100 μM ceramide for 12 h, Relative mRNA expression levels for SERCA2α, SERCA2β and SERCA3 were calculated by normalizing to GAPDH. The figure presents mean fold over control change in experimental groups ± S.D. *P < 0.05 (one-way ANOVA).

The ER is not only responsible for synthesizing and packaging proteins, it also acts as a dynamic Ca^2+^ store. It is well established that disrupting [Ca^2+^]_ER_ homeostasis activates ER stress [21, 22]. To investigate the mechanism of ceramide-induced ER stress, the Ca^2+^-sensitive fluorescent probe Fluo 4-AM was applied to ACCs and visualized using a confocal microscope. Significant elevation of fluorescent intensity was detected after ceramide addition (Figure 4A), indicating the ability of ceramide to induce Ca^2+^ release from the ER to the cytoplasm.

To determine the mechanism of [Ca^2+^]_ER_ depletion, we evaluated changes in SERCA, which pumps cytoplasmic Ca^2+^ to ER lumen. Real-time PCR showed treatment with 100 μM ceramide for 12 h significantly inhibited SERCA2α, SERCA2β and SERCA3 mRNA expression in ACC-2 and ACC-M cells (Figure 4B). These results indicate that ceramide induces [Ca^2+^]_ER_ depletion and disrupts Ca^2+^ homeostasis by inhibiting SERCA expression, thus further increasing ER stress.

### The chemical chaperone TUDCA alleviates ceramide-induced ER stress

**Figure 5:**
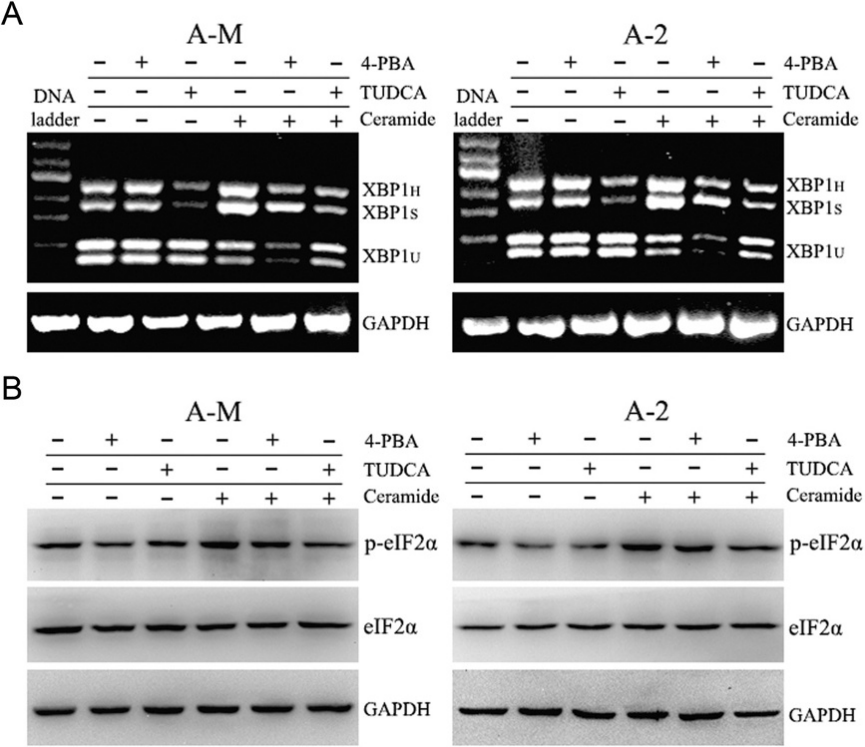
**TUDCA inhibits ceramide-induced ER stress. (A)** ACC-M and ACC-2 cells were seeded into 60 mm culture dishes and the next day cells were pretreated with 5 mM 4-PBA or 1 mg/ml TUDCA. After 3 h incubation, 100 μM ceramide was added for further 12 h incubation in full medium. Total RNA was isolated for detection of XBP1 mRNA splicing using RT-PCR. The experiment was repeated three times and the representative result is shown. **(B)** Cells were treated as in A. After 5 mM 4-PBA or 1 mg/ml TUDCA treatment for 3 h, 100 μM ceramide was added for further 3 h incubation. Whole cell lysates were collected to analyze phosphorylated eIF2α expression. The experiment was repeated several times and the representative result is shown.

It has been reported that chemical or pharmaceutical chaperones, including 4-phenylbutyric acid (4-PBA) and endogenous bile acid derivatives, such as tauroursodeoxycholic acid (TUDCA), alleviate ER stress. To further investigate the mechanism of ceramide-induced ER stress, 5 mM 4-PBA or 1 mg/ml TUDCA was added to ACC cultures 3 h before ceramide treatment. RT-PCR showed that ceramide-induced XBP1 mRNA splicing was significantly inhibited by TUDCA pretreatment, while only slightly inhibited by 4-PBA pretreatment (Figure 5A). Western blot also demonstrated that TUDCA inhibited ceramide-induced eIF2α phosphorylation, while 4-PBA only had a marginal inhibitory effect on ceramide-induced ER stress (Figure 5B). Additionally, we also observed by RT-PCR and western blot that 4-PBA or TUDCA treatment alone inhibited ER stress marker XBP1_S_ and p-eIF2α expression (Figure 5A and B). Overall, these results suggest that the chemical chaperone TUDCA alleviates ceramide-induced ER stress, while 4-PBA does not have a significant inhibitory effect.

### Ceramide induces cell death through ER stress-mediated apoptosis pathway

**Figure 6:**
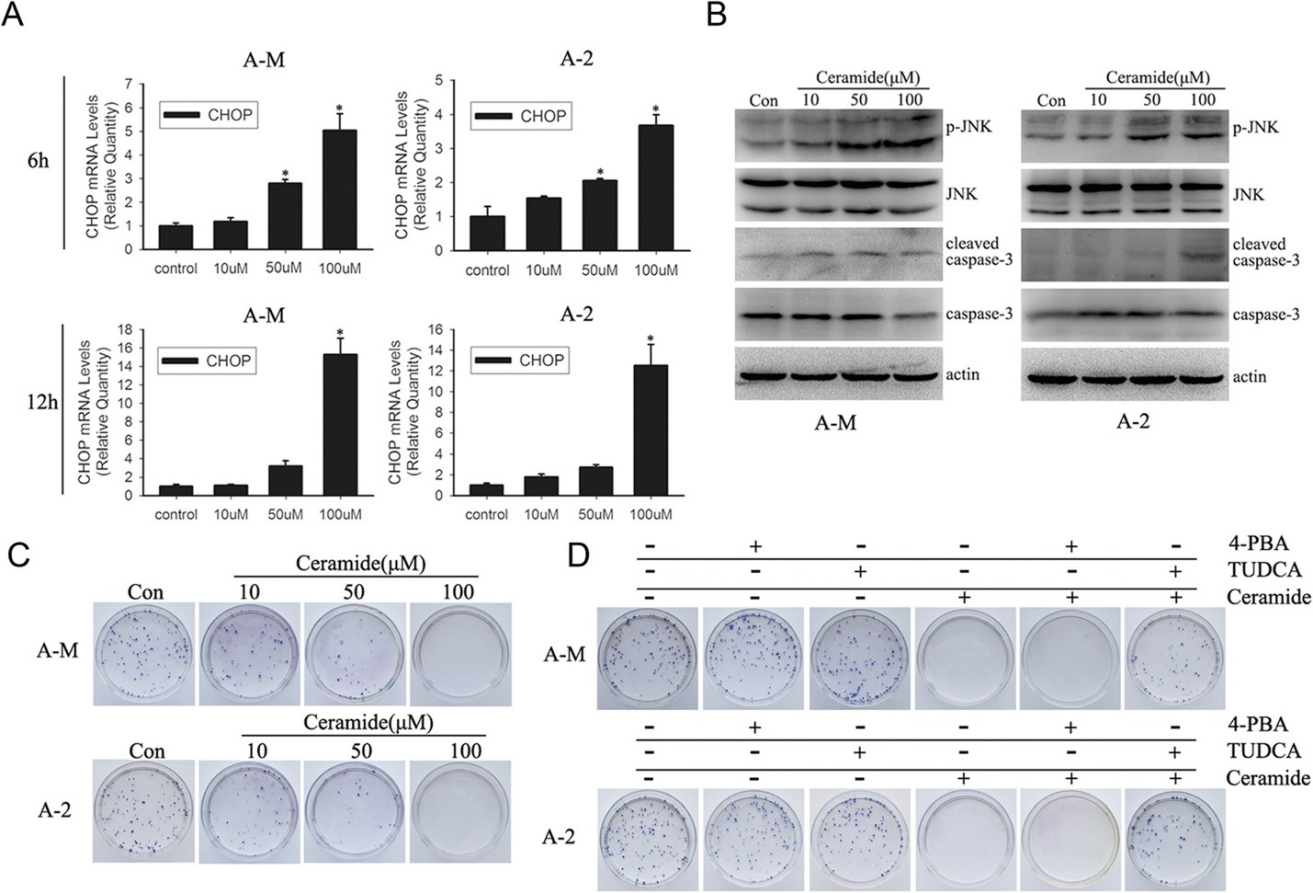
**Ceramide induces ER stress-mediated cell death. (A)** ACC-M and ACC-2 cells were treated with indicated concentration (10–100 μM) of ceramide for 6 and 12 h, total RNA was subjected to Real-time PCR. Relative mRNA expression levels for CHOP were calculated by normalizing to GAPDH. *P < 0.05 (one-way ANOVA) versus control cells. **(B)** Cells were treated as in A. After 12 h treatment with ceramide, whole cell lysates were collected and subjected to western blot. The level of phosphorylated JNK, total JNK, cleaved caspase-3 and total caspase-3 was analyzed with primary antibody. Actin were used as loading controls. The experiment was repeated several times and the representative result is shown. **(C)** ACC-M and ACC-2 cells were seeded into 60 mm culture dishes and the next day cells were treated with indicated concentration (10–100 μM) of ceramide for 12 h and further grown for 3 weeks. Colonies were stained and counted. The experiment was repeated 3 times and the representative result is shown. **(D)** Cells were treated as in C. Cells were pretreated with 5 mM 4-PBA or 1 mg/ml TUDCA. After 3 h incubation, 100 μM ceramide was added for further 12 h incubation in full medium. As showed in images, ER stress inhibitor TUDCA rescued cells from ceramide-induced cell death. The experiment was repeated 3 times and the representative result is shown.

It is well documented that strong or prolonged ER stress leads to cell death. We next investigated the possible link between ceramide-induced ER stress and cell death. CHOP is the central transcription factor upregulated during ER stress and is considered a major trigger of ER stress-mediated apoptosis. Real-time PCR showed increased CHOP mRNA expression after treatment with 100 μM ceramide for 6 h, with prolonged treatment for 12 h further upregulating CHOP expression in ACC-M and ACC-2 cells (Figure 6A). Activation of IRE1α by ER stress induces JNK phosphorylation [23]. Using western blotting, we also observed that treatment with 100 μM ceramide for 12 h significantly induced JNK phosphorylation and increased cleaved caspase-3 expression (Figure 6B). Colony formation assay demonstrated that ceramide induced significant cell death (Figure 6C). Pretreatment of ACC-M and ACC-2 cells with TUDCA, but not 4-PBA, inhibited ceramide-induced cell death (Figure 6D). These results suggest that ceramide triggers cell death by an ER stress-mediated mechanism. Inhibition of ER stress-mediated apoptotic pathway by the ER stress alleviator TUDCA suppresses the cytotoxicity of ceramide.

### ER stress is induced by ceramide independent of its downstream metabolite S1P

**Figure 7:**
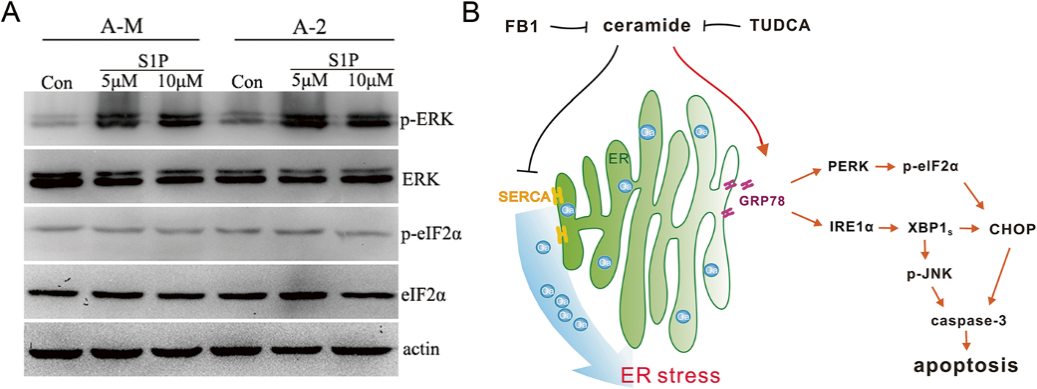
**Ceramide triggers ER stress is independent of its downstream metabolite S1P. (A)** ACC-M and ACC-2 cells were seeded into 60 mm culture dishes and the next day cells were treated with 5–10 μM S1P. The level of phosphorylated eIF2α and ERK was analyzed. Unphosphorylated eIF2α and ERK were measured and actin were used as loading control. The experiment was repeated several times and the representative result is shown. **(B)** Proposed mechanism of ceramide-mediated activation of ER stress response in ACCs. Ceramide induces SERCA inhibition and ER calcium depletion. This leads to increase GRP78 and activate PERK/eIF2α and IRE1α/XBP1 arm of ER stress. FB1 inhibits ceramide-induced ER stress. Prolonged ER stress eventually induces apoptosis through activates pro-apoptotic proteins CHOP and JNK.

Ceramide is catalyzed by ceramidase and sphingosine kinase to produce the downstream metabolite S1P, and treating HL-60 cells with exogenous C2-ceramide increases S1P production [24]. Intracellular and extracellular S1P are both reported to trigger ER stress [25]. We further investigated whether increased levels of the downstream metabolite S1P are responsible for ceramide-induced ER stress. Western blot showed that treatment of ACC-M and ACC-2 cells with 5–10 μM exogenous S1P induced ERK1/2 phosphorylation. However, treatment of the cells with 5–10 μM S1P had no significant effect on eIF2α phosphorylation (Figure 7A). These results indicate that ceramide-induced ER stress is independent of its downstream metabolite S1P.

## Discussion

In this study, we have shown that ceramide induces apoptosis in ACC-M and ACC-2 cells through a novel mechanism involving [Ca^2+^]_ER_ depletion and SERCA inhibition, leading to ER stress and expression of downstream pro-apoptotic factors CHOP and p-JNK. The ceramide synthase inhibitor FB1 and chemical chaperone TUDCA inhibit ceramide-induced ER stress and subsequent cell death. In contrast to ER stress mediated by S1P elevation after SPP1 depletion, ER stress induced by exogenous ceramide is independent of its downstream metabolite S1P. These findings are summarized in Figure 7B. Delineating the ceramide-induced pro-apoptotic signaling cascades will provide potential therapeutic targets for cancer therapy.

Multiple stimuli under physiological or pathological conditions induce the accumulation of unfolded protein in the ER, which activates an evolutionarily conserved adaptive response termed the UPR which leads to cell death if the stimulus is severe or prolonged. The ER chaperone GRP78 acts as a major regulator of the UPR through direct interaction with UPR sensors PERK, IRE1α and ATF6. GRP78 maintains the three sensors in inactive forms under homeostatic conditions, and releasing them for activation upon ER stress. Increased GRP78 expression was observed in ACC-M and ACC-2 cells upon exogenous C2-ceramide treatment, indicating initiation of the ER stress and activation of the UPR cascades. Moreover, ceramide induced the ER stress response in a time- and dose-dependent manner, in support of the statement that the ER stress is increased as the stimulus is intensified and prolonged [1, 15, 19, 21].

In contrast to the selective activation of the ATF6/CHOP pathway of ER stress in response to CerS6/C16-ceramide down-regulation [13, 14], we found in this study that exogenous C2-ceramide treatment induced phosphorylation of eIF2α, suggesting the activation of the PERK/eIF2α arm of the ER stress response. IRE1α is a transmembrane protein that has both a Ser/Thr kinase domain and an endoribonuclease domain. Activated IRE1α uses its endoribonuclease activity to cleave a 26 base intron from XBP1 mRNA, resulting in a translational frameshift and translation of a spliced form of XBP1 (XBP1_S_), which is a more stable and potent transcription factor for target genes involved in protein folding and ER-associated degradation [1, 16]. Increased XBP1_S_ expression was observed in ACC-M and ACC-2 cells upon C2-ceramide treatment, suggesting that ceramide also activates the IRE1α/XBP1_S_ arm. The ceramide synthase inhibitor FB1 is reported to inhibit *de novo* biosynthesis of ceramide [26]. In this study, FB1 treatment abolished ceramide-induced ER stress in ACC-M and ACC-2 cells, whereas interestingly, FB1 alone had no inhibitory effect on the splicing of XBP1 or phosphorylation of eIF2α. Consistently, other researchers reported that FB1 treatment alone does not impair the splicing of XBP1 in LPS-treated B cells or XBP1-deficient B cells [27]. It might be due to the relatively low level of endogenous ceramide expression in ACC-M and ACC-2 cells, mitigating the inhibitory effect of FB1 on ceramide-induced ER stress response.

The ER is the major intracellular Ca^2+^ store, and perturbation of [Ca^2+^]_ER_ homeostasis has been reported to induce ER stress. Ca^2+^ is pumped from the cytosol to the ER by SERCA and released through either the inositol-1,4,5-trisphosphate receptor/Ca^2+^ channels or ryanodine receptor/Ca^2+^ channels [28–30]. Although alteration of endogenous C16-ceramide levels by CerS6 knockdown has been reported previously to trigger ER stress by modulating SERCA expression and subsequently changing the [Ca^2+^]_ER_/[Ca^2+^]_IN_ ratio [14], data presented here are novel because the role of exogenous ceramide in the induction of [Ca^2+^]_ER_ depletion by SERCA2/3 inhibition have not been described previously. Our data showed that exogenous ceramide treatment disrupts Ca^2+^ homeostasis by inducing [Ca^2+^]_ER_ depletion, which is in agreement with previous reports that release of [Ca^2+^]_ER_ and the subsequent increase of Ca^2+^ concentration in the cytosol and mitochondrial matrix play an important role in exogenous ceramide-induced apoptosis [31, 32].

Both 4-PBA and TUDCA have been reported to alleviate ER stress, but by distinct mechanisms. Recent studies suggest that 4-PBA represses ER stress by stabilizing protein conformation in the ER [33–37], while TUDCA reduces [Ca^2+^]_IN_ concentration after Thapsigargin treatment, thus inhibiting ER stress and apoptosis [38]. TUDCA was reported to be more effective in inhibiting ER stress and protecting ER stress-mediated apoptosis than 4-PBA in steatotic and non-steatotic livers during partial hepatectomy under ischemia-reperfusion [35]. In the present study, we observed that 4-PBA or TUDCA treatment alone reduced XBP1_S_ and p-eIF2α expression, whereas TUDCA had more profound effects on impairing ceramide-induced ER stress than did 4-PBA, and only TUDCA is effective in inhibiting ceramide-induced cell death. These results suggest that exogenous ceramide triggers ER stress and apoptosis through mechanisms that can be largely inhibited by TUDCA. Recent studies suggest that both Ca^2+^ overload and [Ca^2+^]_ER_ depletion result in changes in protein folding and ER stress [22, 39, 40]. Based on our findings and the mechanism by which TUDCA alleviate the ER stress, we speculated that ceramide-induced [Ca^2+^]_ER_ depletion might play a major role in pro-apoptotic mechanisms in ACC-M and ACC-2 cells.

Activation of CHOP is a common point of convergence for all three arms of the UPR. Up-regulated ATF6, ATF4 or XBP1_S_ expression induces apoptosis by interacting with binding sites within the promoter of the CHOP gene. In addition to mediating the down-regulation of Bcl-2 and up-regulation of Bim, CHOP also induces expression of the pro-apoptotic proteins ERO1α and Puma [1–3, 19, 21]. Pro-apoptotic ER stress eventually leads to mitochondria dysfunction, cytochrome *c* release and caspase-3 cleavage [10, 41]. It has been demonstrated that reduction of C16-ceramide by CerS6 knockdown activates CHOP expression [13]. Our study shows for the first time that exogenous short chain ceramide activated CHOP expression in a time- and dose-dependent manner via induction of ER stress. We also identified activation of JNK and elevated expression of cleaved caspase-3 in ceramide-treated ACCs. These findings suggest that exogenous ceramide definitely activates the ER stress-mediated pro-apoptotic signaling pathways, and promotes the commitment phase of apoptosis.

Ceramide and its downstream metabolite S1P have long been reported to play opposing roles in the regulation of autophagy, angiogenesis and senescence. A recent report demonstrates that elevated intracellular S1P owing to S1P phosphohydrolase 1 depletion significantly activates ER stress and survival signaling via the Akt pathway [25]. Our data showed that exogenous S1P treatment had no significant effect on ER stress, which suggests ER stress triggered by ceramide is independent of S1P. It might be interesting to further determine how the metabolic interconversion of ceramide and S1P regulates ER stress.

## Materials and methods

### Cell culture

Human salivary adenoid cystic carcinoma (ACC-M and ACC-2) cell lines were purchased from China Center for Type Culture Collection (Wuhan, China). Cells were cultured in DMEM containing 10% fetal bovine serum and antibiotics and maintained in a humidified chamber (5% CO_2_/95% air) at 37°C.

### Chemicals and reagents

C2-ceramide (Avanti Polar Lipid, Alabaster, AL, USA), TUDCA (Calbiochem, EMD-Millipore, Billerica, MA, USA), Tunicamycin, Thapsigargin, FB1, 4-PBA, S1P, Pluronic F-127 (Sigma-Aldrich, St Louis, MO, USA), and Fluo 4-AM (Dojindo Laboratories, Kumamoto, Japan) were reconstituted as recommended by their respective manufacturers. Antibodies against p-eIF2α (Ser51), eIF2α, JNK, cleaved caspase-3, p-ERK (Thr202/Tyr204), ERK (Cell Signaling Technology, Beverly, MA, USA), p-JNK (Thr183/Tyr185) (Abcam, Cambridge, MA, USA), caspase-3 (Abgent, San Diego, CA, USA), and actin (Santa Cruz, Dallas, TX, USA) were used in this study. All secondary antibodies were purchased from Abcam.

### RT-PCR and Real-time PCR

Cells were rinsed with PBS and lysed in Trizol (Invitrogen, Carlsbad, CA, USA) and 1 μg of total RNA was used for cDNA synthesis using PrimeScript™ RT reagent kit with gDNA Eraser (Takara Bio, Tokyo, Japan). RT-PCR for XBP1 and glyceraldehyde 3-phosphate dehydrogenase (GAPDH) was performed using Takara Ex Taq™ polymerase. The amplicons of XBP1 were digested by PstI restriction enzyme and resolved using a 2% agarose gel. Real-time PCR was carried out using gene-specific primers (Table 1), cDNAs, QuantiFast SYBR Green PCR Kit (Qiagen, Hilden, Germany), and an Applied Biosystems 7300 Real-Time PCR System (Applied Biosystems, Foster City, CA, USA) according to the manufacturer’s instructions. The details of the primers for each gene are given in Table 1. Analysis of RT-PCR and Real-time PCR results was performed after normalizing to GAPDH.

**Table 1:** **Sequences of primers used in RT-PCR and Real-time PCR**

Gene	Forward primer sequence (5′–3′)	Reverse primer sequence (5′–3′)
XBP1	CCTTGTAGTTGAGAACCAGGAG	GGTCCAAGTTGTCCAGAATGC
GAPDH	AGGTCCACCACTGACACGTT	GCCTCAAGATCATCAGCAAT
XBP1_S_	CCGCAGCAGGTGCAGG	GGGGCTTGGTATATATGTGG
XBP1_t_	CCTTGTAGTTGAGAACCAGG	GGGGCTTGGTATATATGTGG
GRP78	TTCTTGTTGGTGGCTCGACT	GTCAGCATCTTGGTGGCTTT
CHOP	AGGCACTGAGGGTATCATGTT	CTGTTTCCGTTTCCTGGTTC
SERCA2α	CTGTCCATGTCACTCCACTTCC	AGCGGTTACTCCAGTATTGCAG
SERCA2β	TCATCTTCCAGATCACACCGC	GTCAAGACCAGAACATATC
SERCA3	CACCAGCCCTGAAGAAAGCA	AGGAGATGAGGTAGCGGATGAAT

### Western blot

Cells were rinsed with PBS and harvested in lysis buffer containing 20 mM HEPES (pH 7.4), 1% Triton X-100, 10% glycerol, 2 mM EGTA, 1 mM sodium vanadate, 2.5 mM sodium pyrophosphate, 25 mM sodium glycerophosphate, 50 mM NaF, complete EDTA-free protease inhibitor cocktail and the phosphatase inhibitor cocktail PhosStop (Roche, Mannheim, Germany). Equivalent amounts of protein (40–100 μg) were subjected to SDS-PAGE and transferred to polyvinylidene difluoride (PVDF) membrane (Millipore, Bedford, MA, USA). After blocking with 10% non-fat dry milk or BSA for 1 h, membranes were incubated with specific antibodies overnight at 4°C, followed by incubation with secondary antibodies for 1 h at room temperature. Proteins were detected using horseradish peroxidase-conjugated secondary antibodies and an enhanced chemiluminescence reagent (Millipore). The intensity of each band was quantified using Quantity One software (BioRad, Hercules, CA, USA) after normalization to corresponding loading controls.

### Intracellular Ca^2+^ measurement

Cells were seeded on a confocal dish with a glass bottom. The cells were loaded with dye by incubating with 5 μM Ca^2+^-sensitive probe Fluo 4-AM in the presence of 0.05% Pluronic F-127 in HBSS for 30 min at 37°C, and then washed three times with HBSS to remove the extracellular Fluo 4-AM and incubated in HBSS containing 1% FBS for 20 min at 37°C. Cells were treated initially with HEPES buffer and then with buffer containing 100 μM C2-ceramide. Changes in fluorescent intensity were monitored using an Olympus FluoView FV1000 confocal laser scanning microscope (Olympus, Tokyo, Japan). Image was analyzed by Olympus FV10-ASW 3.1 Viewer software, using Time-series mode.

### Colony formation assay

Cells were seeded into 60 mm culture dishes at 200 cells per dish. After 24 h, cultures were replaced with fresh medium containing 10% FBS with or without 10–100 μM ceramide. For ER stress inhibition, cells were pretreated for 3 h with 5 mM 4-PBA or 1 mg/ml TUDCA prior to ceramide addition. After 24 h incubation, culture dishes were rinsed three times with PBS. Cells were further grown in fresh medium containing 10% FBS for 3 weeks. Colonies were stained for 15 min with a solution containing 0.5% crystal violet and 25% methanol, followed by three rinses with tap water to remove excess dye. Colonies were counted only if a single clone contained more than 50 cells.

### Statistical analysis

Statistical analysis was performed using SPSS 13.0 (Chicago, IL, *USA*). Statistical analyses were performed using the Student’s t-test or ANOVA for two-way analysis of variance. *P*-values of *P* <0.05 were defined as statistically significant.

## Abbreviations

ER: Endoplasmic reticulum
UPR: Unfolded protein response
SERCA: Sarcoplasmic/endoplasmic reticulum Ca^2+^- ATPase
FB1: Fumonisin B1
[Ca^2+^]_ER_: Endoplasmic reticulum calcium
[Ca^2+^]_IN_: Intracellular calcium
XBP1_S_: Spliced X-box binding protein 1
XBP1_U_: Unspliced X-box binding protein 1
XBP1_t_: Total X-box binding protein 1
eIF2α: Eukaryotic initiation factor 2α
CHOP: CCAAT/enhancer-binding protein (C/EBP) homologous protein
JNK: c-Jun N-terminal kinase
4-PBA: 4-phenyl butyric acid
TUDCA: Tauroursodeoxycholic acid
S1P: Sphingosine-1-phosphate
TG: Thapsigargin
Tm: Tunicamycin.
